# Searching for new loci and candidate genes for economically important traits through gene-based association analysis of Simmental cattle

**DOI:** 10.1038/srep42048

**Published:** 2017-02-07

**Authors:** Jiangwei Xia, Huizhong Fan, Tianpeng Chang, Lingyang Xu, Wengang Zhang, Yuxin Song, Bo Zhu, Lupei Zhang, Xue Gao, Yan Chen, Junya Li, Huijiang Gao

**Affiliations:** 1 Institute of Animal Science, Chinese Academy of Agricultural Science, Beijing, China

## Abstract

Single-marker genome-wide association study (GWAS) is a convenient strategy of genetic analysis that has been successful in detecting the association of a number of single-nucleotide polymorphisms (SNPs) with quantitative traits. However, analysis of individual SNPs can only account for a small proportion of genetic variation and offers only limited knowledge of complex traits. This inadequacy may be overcome by employing a gene-based GWAS analytic approach, which can be considered complementary to the single-SNP association analysis. Here we performed an initial single-SNP GWAS for bone weight (BW) and meat pH value with a total of 770,000 SNPs in 1141 Simmental cattle. Additionally, 21836 cattle genes collected from the Ensembl Genes 83 database were analyzed to find supplementary evidence to support the importance of gene-based association study. Results of the single SNP-based association study showed that there were 11 SNPs significantly associated with bone weight (BW) and two SNPs associated with meat pH value. Interestingly, all of these SNPs were located in genes detected by the gene-based association study.

Carcass and meat-quality traits have attracted much attention from breeders in the beef cattle industry. Meat-quality traits are mainly measured by composition, quality, and palatability factors such as visual appearance, smell, firmness, juiciness, tenderness, and flavor. Some researchers find that meat pH value is highly correlated with other meat-quality measurements (e.g. drip loss and texture score) and carcass yield (e.g. carcass weight, loin depth, loin length)^1^. Improving the meat-pH value has become a high priority for the beef industry to satisfy consumer preferences. On the other hand, the proportion of genetic variation explained by single-nucleotide polymorphism (SNP) based genome-wide association study (GWAS) of bone weight (BW) and pH value are often significantly lower than the heritability estimates for the traits^2^. For example, the heritability for BW is as high as 41% in our analysis. However, the 12 genetic loci identified for BW to account for only ~4.2% of the phenotypic variance in BW, which means that many genetic variants with smaller effects failed to be detected by GWAS. Therefore, association analysis of complex traits for BW and pH value in Simmental cattle is not sufficient, and further study is required to detect more loci.

It is well known that traditional GWAS is an individual–marker-based analysis that has been very successful in identifying disease loci in humans and economically important traits in domestic animals^3^,^4^,^5^. However, single-SNP analysis often focuses on only a few of the most significant SNPs in the genome, and these loci only explain a small proportion of the genetic risk for diseases or complex traits^6^,^7^. This limitation may be improved by employing a gene-based GWAS analytic approach. A gene-based association analysis can combine genetic information for all SNPs in a gene, increase the capability to find novel genes, and generate more informative results.

Different approaches have been used to identify genes that are associated with traits of interest^8^,^9^,^10^. One of the best known gene-based algorithms is the Gene-based Association Test using Extended Simes (GATES) method, which combines the p values of the SNPs within a gene to obtain an overall p value for the association of the entire gene^9^. This method does not consider other factors, such as gene size and linkage disequilibrium (LD) between markers. As a result, it often produces more false discoveries. Another well-known gene-based GWAS algorithm was proposed by Capomaccio *et al*. in 2015, and this method uses the Multiple Species Gene-Based Association Suite (MUGBAS) for discrete traits and a set-based test for discrete and continuous traits^10^. Nonetheless, the set-based test requires heavy computation, therefore, limits its application at a genome-wide level. An efficient genome-wide gene-based association method was developed, we performed a modified gene-based analysis for GWAS studies^11^. For a given gene contains several SNPs, we first used principal component analysis (PCA) to extract PCs, and then ranked all of these PCs based on the significance of their statistical association with a trait of interest. Finally, we calculated the gene’s statistical value using Fisher’s combination test for gene association^12^. This procedure was used to test whether the set of genes was significantly associated with the traits of interest. In this study, we focused on genes associated with the traits of BW and meat pH value.

## Materials and Methods

### Ethics statement

The study was approved by the Science Research Department of the Institute of Animal Science, Chinese Academy of Agricultural Sciences (CAAS) (Beijing, China). All procedures were in strict accordance with the guidelines proposed by the China Council on Animal Care. Using animals and private land in this study were approved by the respective owners.

### Animal resources and phenotypes

We established the Simmental cattle population in the Ulgai, Xilingol league, Inner Mongolia, China. The population consisted of 1141 young Simmental cattle born between 2009 and 2014. After weaning, the cattle were transferred to the Jinweifuren cattle farm (Beijing) for fattening in a uniform feeding and management environment. The cattle were observed for growth and developmental traits until slaughter at 16–18 months of age. Our study focused primarily on phenotypic traits associated with carcass quality and meat quality; therefore, during the period of slaughter, we measured the traits in strict accordance with the guidelines proposed by the Institutional Meat Purchase Specifications for fresh beef. First, this study was performed on the trait bone weight (BW), which was measured in half of the cattle carcass. After removing the exposed meat from the bone, the weight of the remaining bone was defined as BW. For the pH value, we used a steak from the twelfth rib at slaughter and measured pH at three locations using a Mettler Toledo pH meter (Mettler Toledo, Greifensee, Switzerland). Summary statistics of the two traits were given in Table 1.

### Genotyping and quality control

Blood samples were collected along with the regular quarantine inspection of the farms. Genomic DNA was extracted from blood samples using a TIAN amp Blood DNA Kit (Tiangen Biotech Company Limited, Beijing, China), and DNA with an A260/280 ratio ranging between 1.8 and 2.0 was subjected to further analysis. The Illumina Bovine HD Bead Chip contained 774,660 SNPs was used for individual genotyping. The SNPs were uniformly distributed across the whole bovine genome. The average distance between consecutive markers is 3.43 Kb with a standard deviation of 4.38 Mb. The genotyping platform adopted in this study was Illumina (San Diego, CA, USA) Infinium II Assay. Samples were genotyped using Illumina BEADSTUDIO (Inc.9885 Towne Centre Drive, San Diego, CA 92121 USA) and SNP chips were scanned using Infinium Genome Studio.

Regarding quality control, we used the PLINK software (v1.9, http://pngu.mgh.harvard.edu/~purcell/plink/) to remove individuals and SNPs based on the following criteria. All markers with call rates <90%, minor allele frequencies (MAFs) <5%, occurrence of the genotype in <5 individuals, or severe departure from Hardy-Weinberg equilibrium (with p < 10-6) were excluded from the analysis. All animals with missing genotypes >10% or SNP Mendel error rate >2% were removed from the data. In addition, all SNPs with uncertain map positions were excluded from the analysis. Ultimately, a total of 1141 individuals and 677,855 SNPs remained in the final data for the subsequent analysis.

## Gene-based association analysis

### Phenotypic preparation

Before the association study, phenotypic values of the two traits were adjusted by effects of year, farm, gender, fattening days (the date since entering fattening farm to slaughtering), entering weight (live weight when entering fattening farm). In additional, we also used the corresponding population structure matrix constructed by the first five PCs from a portion of SNPs over the bovine genome to correct the population structure (similar with the PCs calculation in single marker GWAS), using a general linear model. In other words, the residuals after fitting the above fixed effects were treated as the phenotypic values of traits for the association studies.

### Gene collection and definition of gene locus

One of the major steps of a gene-based analysis is the assignment of SNPs to genes. All genes including coding sequences, non-coding sequences and pseudogenes, were defined based on the Bos taurus UMD 3.1 Sequence. Sequences were all downloaded from the Ensembl Genes 83 database in the BioMart (http://asia.ensembl.org/biomart/martview). SNPs were mapped to genes according to their physical distance: a SNP was mapped to the gene whose coding sequence had an overlap with a 20 kb range around the gene^13^. Only genes with at least five mapped SNPs were included in the analysis. Ultimately, a total of 21836 genes was mapped onto the downloadable genes in this analysis.

### Two-stage-gene-based association study

#### Stage 1: PC construction within a gene

In the first stage, we constructed principal components (PCs) within each gene. We treated each SNP within a gene as a variable and calculated the variance-covariance matrix for all SNPs. We then calculated the eigenvalues and eigenvectors of the covariance matrix. The PCs were obtained by multiplying the eigenvectors by the SNP genotype matrix. Finally, we selected the top PCs that contributed 85% of the total variation of SNP data. These PCs were treated as the independent variables for subsequent regression analyses.

#### Stage 2: Fisher’s combination test for each gene

In the second stage, the PCs within each gene were treated as independent variables for regression analysis and significance tests. The response variables were the two traits after correction for the fixed effects. Because the PC’s were independent, the test for association of a trait and a PC was done independently. The simple correlation coefficient for the corrected phenotypes and PCs could be used to calculate the p values of PCs.

In the Fisher’s combination test for each gene, *χ*^2^ was constructed by combining K independent p values for PCs, as follows:


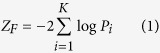


Under the null model, this test statistic followed a *χ*^2^ distribution with degree of freedom 2 K. A new p-value for the gene was calculated from this Chi-square distribution.

Bonferroni correction was adopted for multiple tests of all genes in the genome. A gene was considered significant at the genome-wide significance level if the nominal p value was less than 0.05/N. For the gene-based association analysis, a total of 21836 genes was mapped onto the available genes in this analysis. We therefore chose N = 21836 and defined the gene-based genome-wide significance level as 0.05/21836 = 2.29 × 10−6.

### Single-marker association analysis

The compressed mixed linear model (CMLM) was used cluster analysis to assign similar individuals to the same groups and fit the “Q + K” matrix into the model to improve statistical power^14^. We used CMLM to detect individual markers using the default parameters provided by the Genome Association and Prediction Integrated Tool (GAPIT)^15^. First, a principle components analysis (PCA) was performed and a kinship matrix was calculated using the GAPIT package in R. The “Q” matrix was determined by the PCA to account for effects due to population structure, and the kinship matrix (K) was calculated using the VanRaden algorithm^16^ to replace the incomplete pedigrees. To avoid the bias caused by potential LD and reduce computing time, a set of SNPs were randomly selected with ‘SNP.fraction’ as input parameter for the PCs calculations^15^. The fraction of SNP number can be controlled by “Ratio” parameter in GAPIT software. We set SNP.fraction to 10%, and finally correct the population structure using the first five PCs estimated from a portion of SNPs. For each trait, the model was


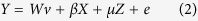


where *Y* is the vector of phenotypic value; v is the vector of unknown fixed effect of the current marker; *β* is the vector of fixed effects, including years, farms, gender, fattening days, entering weight, and PCs; *μ* is a vector of random additive genetic effects corresponding to the clustered groups with an assumed N(0, *σ*^2^*K*) distribution, where *σ*^2^ is the additive genetic variance and *K* is the compressed kinship matrix; *W* is a vector of the SNP genotype indicators and has a value of 0, 1, or 2, corresponding to AA, AB, BB (B being the minor allele); *X* and *Z* are the incidence matrices for *β* and *μ*, respectively; and *e* is a vector of random residual effects with an assumed N(0, 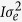), where 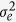 is the residual error variance. For each SNP, a *t*-test was used to examine the association between the SNP and the trait.

Quantile-Quantile (Q-Q) plots were used to assess how well the model captured population structure and familial relatedness. In the Q-Q plot, the y-axis represented the negative logarithms of the p values from the models, and the x-axis represented the expected value under the null hypothesis (SNPs didn’t associate with the traits). Because a total of 677,855 SNPs was used for this analysis, the number of potential associated SNPs after Bonferroni correction (0.05/677855 = 7.38 × 10-8) was too small, resulting in very low statistical power^17^. Therefore, a suggestive significance threshold for p value (1/677855) = 1.47 × 10-6) was used in this analysis^18^,^19^,^20^.

## Results

### Population stratification assessment

**Figure 1:**
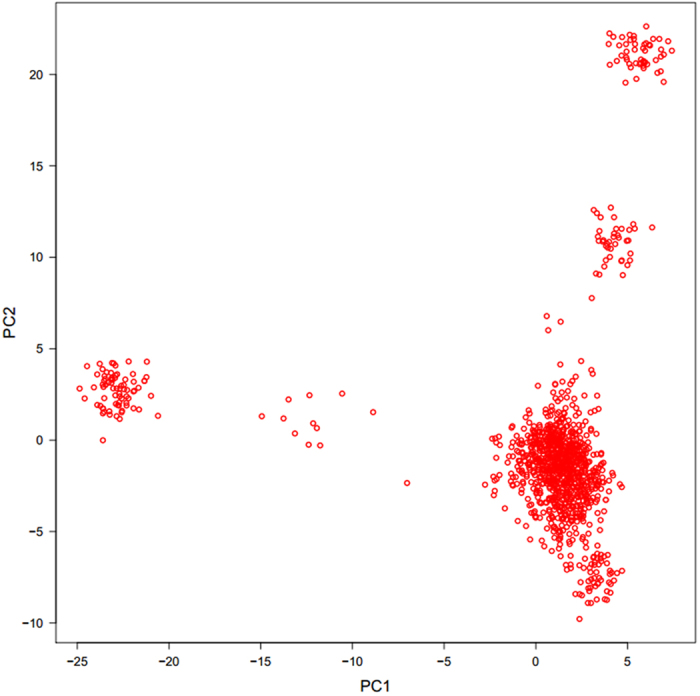
Principal components (PC) plot. The second principal component (PC2) plotted against the first principal component (PC1).

Figure 1 shows the population stratification of the Simmental population. The PCA result shows five separate clusters. Population stratification due to different genetic backgrounds and farms was considered as an underlying confounder in the association analysis.

### Significant SNPs

**Figure 2:**
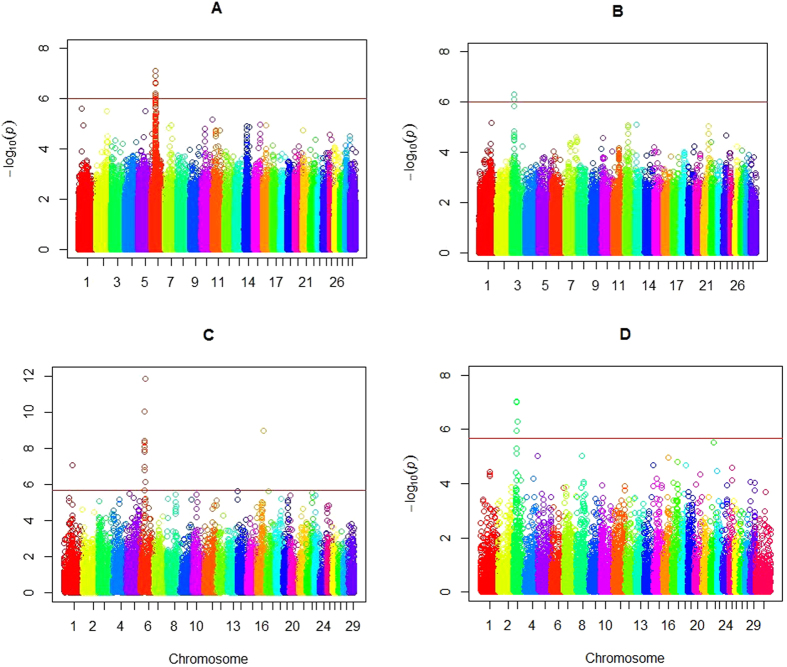
Manhattan plots of −log10(*p* values) for two traits from the single-SNP method and the gene-based method. Panels A and B are the plots for bone weight and pH values, respectively, in the single-marker analysis. Panels C and D are the plots for bone weight and pH values, respectively, in the gene-based association analysis. The 29 chromosomes are color coded. The red horizontal line indicates genome-wide significance level from Bonferroni correction (−log10(1/677855)) for the single-marker analysis. The genome-wide significance level for the gene-based method is (−log10 (0.05/21836)).

**Figure 3:**
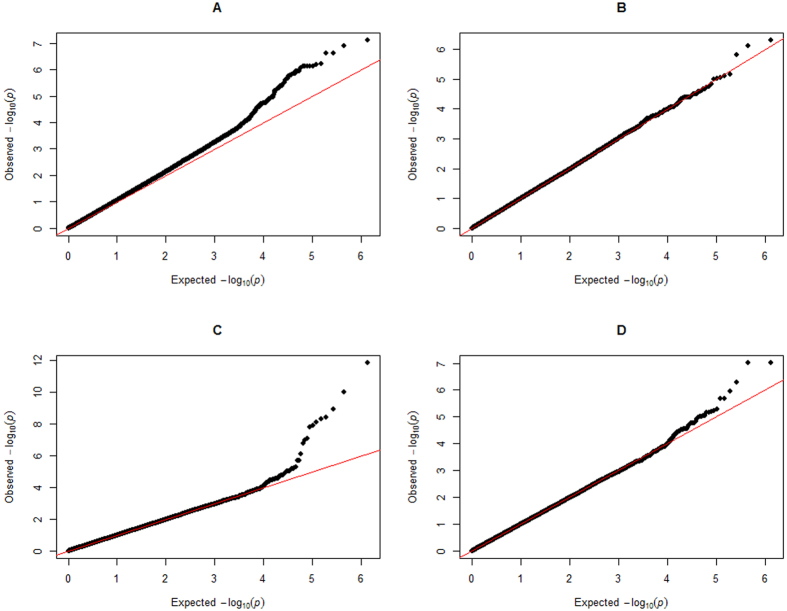
The Quantile-Quantile plot of p-values. The observed negative logarithms of the p values in GWAS using the gene-based method and the single-marker method for two traits are plotted against their expected values under the null hypothesis of no association with the trait. Panels A and B show the plots for bone weight and pH value, respectively, in the single-marker analysis. Panels C and D show the plots for bone weight and pH value, respectively, in the gene-based association analysis.

Significant SNPs were shown in Table 2, which also included Bos Taurus Autosome, SNP position in the genome, nearest known gene, MAFs, and p value (<1.47 × 10-6). Figure 2A and B shows the Manhattan plots for all SNPs. To summarize, the number of significant SNPs identified by CMLM was 11 for BW and 2 for pH value. For BW, most of the significant SNPs were located in or near *LAP3, LCORL, FAM184B*, and *NCAPG* on BTA6. The two SNPs significant for pH value were located near *S100A10* on BTA3. The Q-Q plots for the two traits (Fig. 3A and B) suggested that there was no inflation or systematic bias in this study. Most of the points were concentrated along a diagonal line because the GWAS model sufficiently accounted for the population structure, and only a small number of SNPs were associated with the traits.

### Significant Genes

For the gene-based association analysis, the Manhattan plots were shown in Fig. 2C for BW and Fig. 2D for pH value. Table 3 displays the significant genes detected by the gene-based method, including their starting and ending positions in the genome, the ensemble IDs, Bos Taurus Autosome, and the p values. The total number of significant genes identified by the gene-based GWAS was 12 for BW and 3 for pH value. For trait BW, a total of 10 detected genes were located on BTA6, 1 was located on BTA1, and 1 was located on BTA16, and four already detected genes (*LAP3, LCORL, FAM184B, and NCAPG*) on BTA6 in the gene-based association analysis were all found in the single-marker analysis. For pH value, we found 3 genes, all located on BTA3. Similar to the single-marker analysis, we found one already detected gene (*S100A10*) on BTA3 in the gene-based association. In summary, most of the significant SNPs detected by the individual-SNP analysis were also detected in the gene-based association analysis. The Q-Q plots for the test statistics of the gene-based analysis are shown in Fig. 3C and D for BW and pH value, respectively.

## Discussion

In this study, a gene-based association strategy was used to identify new associations of genetic variants with BW and meat pH value in Simmental cattle. The gene-based association analysis combined genetic information given by all the single nucleotide polymorphisms (SNPs) in a gene. Our method orthogonalizsed the SNPs within each gene using PCA so that the Fisher’s method can be used to calculate a new p-value for a gene from p-values of multiple independent PCs. Of course, choosing PCs to formulate the statistic for a gene takes advantage of information on those SNPs. Therefore, the gene-based method can increase the probability to find novel genes, and generate more informative results than the single-marker method. While using the single marker GWAS, none of the SNPs reached the genome-wide significance threshold due to the stringent criterion, which forced us to lower the threshold to pick some of the significant SNPs.

In addition to identify additional loci using the gene-based association analysis approach, we confirmed previously reported associations with genes related to BW. There were a large number of significant SNPs associated with BW located on BTA6^21^,^22^. Similar to the single-marker results, four already detected genes (*LAP3, LCORL, FAM184B, and NCAPG*) on BTA6 were also found in the gene-based association analysis for the trait of BW. The genes *LAP3, LCORL*, and *FAM184B* have been shown to be candidate genes for carcass or growth traits in cattle^23^,^24^,^25^, the most significant SNP (Hapmap26308-BTC-057761) located within LAP3 was also found in black cattle as the second strongest association^26^. In addition, some significant SNPs were in the genes *NCAPG, LAP3 and LCORL*. For *NCAPG*, it encodes a non-structural maintenance of chromosomes (-SMC) condensin I complex and leads to the change of amino acid Ile442 to Met442, which is associated with foetal growth and carcass weight in cattle^25^,^27^,^28^. In addition, the polymorphism in NCAPG shows significant associations with carcass weight, carcass yield estimate, and lipid deposition and has been identified as a candidate causative variant for bovine carcass weight quantitative trait locus (QTL)^23^,^27^,^29^,^30^,^31^. For *LAP3*, it participates in oxytocin hydrolysis and acts as promoter polymorphism and milk production, and is highly expressed in skin and mammary and adipose tissue^23^,^32^,^33^. For *LOCAL*, it encodes a ligand-dependent nuclear receptor co-repressor, may affect skeletal frame size and adult height in human, horse, and cattle^34^,^35^,^36^, and has a strong correlation with the nearest gene *NCAPG*. The *LCORL/NCAPG* locus has been associated with mammalian stature^36^, direct calving ease, feed intake, gain, meat and carcass traits across multiple breeds of cattle^23^,^24^,^37^. Snelling and collaborators detected a 0.57-Mb (37.96 Mb to 38.53 Mb) segment in *LCORL/NCAPG* locus on BTA6 associated with feed intake, gain, meat and growth traits in a crossbred population of beef cattle. In addition, *LAP3, NCAPG*, and *LCORL* have been considered potential positional and functional candidate genes for direct calving ease, lean growth, and fat deposition^24^,^27^. Overall, we suggest a 2.6-Mb (37.3 Mb–39.9 Mb) segment on BTA6 as a candidate region for BW. Similar to the results of single-marker analysis, we found a common gene (S100A10) for the trait pH value with the gene-based association analysis. S100A10 encodes S100 calcium-binding protein A10, which plays a role in calcium ion binding and ion channel binding^38^. S100A10 induces the dimerization of ANXA2/p36, and it may function as a regulator of protein phosphorylation in that the ANXA2 monomer is the preferred target (*in vitro*) of tyrosine-specific kinase^39^. Some researchers found that S100A10 is associated with residual feed intake in Angus cattle^40^. Relevant to our research, we found that it is also associated with the meat trait marbling score in Simmental beef cattle^41^. This gene is thought to be involved in regulation of cell cycle progression and differentiation. It has been implicated in major depression (downregulated in depressed humans and in animal models), suicide (downregulated in peripheral blood of attempters and in prefrontal cortex of suicide completers), and bipolar disorder (upregulated in peripheral blood)^42^,^43^,^44^. In Table 4, for the remaining associated genes, we provided the gene symbols, a brief description of the potential relevance of the gene/gene product to BW and pH based on its function and relevant references.

The gene-based association study may have its own limitations. For example, if a gene contains only one causal variant, inclusion of a large number of null SNPs in the gene-based method may dilute this gene’s significance. In addition, the ±20 kb boundaries defined for a gene are based on prior reports^45^. This way of defining the gene boundaries is quite subjective. Inadaptable boundaries may occur so that some SNPs may be included in multiple genes. It may also be difficult to definitively identify the causal gene when multiple adjacent genes are statistically significant. For these reasons, the gene-based method may not be seen as a replacement for the traditional single-marker analysis, but rather as complementary to it.

In summary, we applied a gene-based association analysis that is complementary to GWAS and identified important genes associated with traits BW and pH value in beef cattle. A series of bioinformatics analyses provide supportive evidence that the gene-based association analysis is useful. We believe that subsequent studies, including further exploration and analysis of these genes, may reveal more causal networks underlying carcass traits in cattle.

## Additional Information

**How to cite this article**: Xia, J. *et al*. Searching for new loci and candidate genes for economically important traits through gene-based association analysis of Simmental cattle. *Sci. Rep.* **7**, 42048; doi: 10.1038/srep42048 (2017).

**Publisher's note:** Springer Nature remains neutral with regard to jurisdictional claims in published maps and institutional affiliations.

## Figures and Tables

**Table 1:** Descriptive statistics of two cattle economical traits.

Trait	Mean	Standard deviation	Maximum	Minimum
BW/kg	40.00	6.58	80.00	19.00
pH	5.63	0.38	7.16	4.00

**Table 2:** Significant SNPs identified for BW and pH value traits by single-marker GWAS method (p < 1.47 × 10^−6^).

SNP	BTA	Position	P value	MAF	Nearest Gene Name	Distance
BovineHD0600010713^a^	6	38686919	2.36E-07	0.3623	FAM184B	14613
BovineHD0600010716^a^	6	38704872	8.77E-07	0.3660	FAM184B	32566
Hapmap26308-BTC-057761^a^	6	38576012	7.84E-08	0.3836	LAP3	in
Hapmap30134-BTC-034283^a^	6	38464203	2.48E-07	0.2661	LAP3	110456
BovineHD0600010646^a^	6	38468901	6.22E-07	0.1321	LAP3	105758
BovineHD4100004580^a^	6	38852093	1.24E-07	0.3952	LCORL	in
BovineHD0600010755^a^	6	38866381	7.34E-07	0.3672	LCORL	in
BovineHD4100004575^a^	6	38830725	7.34E-07	0.3672	LCORL	13269
BovineHD4100004577^a^	6	38837159	7.34E-07	0.3672	LCORL	6835
BovineHD4100004578^a^	6	38840174	7.34E-07	0.3672	LCORL	3820
BovineHD0600010736^a^	6	38762470	1.42E-06	0.3172	NCAPG	3544
BovineHD030000648^b^	3	18808560	4.96E-07	0.3856	S100A10	in
BovineHD0300006049^b^	3	18812141	7.81E-07	0.2458	S100A10	1843

SNP = SNP name in BovineHD panel.

BTA = Bos Taurus Autosome.

Position = position (bp) on UMD3.1 P value = p-values calculated by CMLM.

MAF = Minor Allele Frequency.

Nearest Gene Name = name of the nearest gene.

Distance = distance between SNP and nearest gene.

Note: SNP with a superscript “a” was identified by the trait of BW only, SNP with a superscript “b” was identified by the trait of pH only. And similar with Tables 3 and 4.

**Table 3:** Significant genes identified for BW and pH value traits by gene-based GWAS method.

Ensemble ID	BTA	Start(bp)	End(bp)	P value	Gene symbol
ENSBTAG00000005108^a^	6	41236589	41640789	1.41E-12	SLIT2
ENSBTAG00000021582^a^	6	38765969	38812051	9.60E-11	NCAPG
ENSBTAG00000018303^a^	16	59132781	59456250	1.12E-09	PAPPA2
ENSBTAG00000046561^a^	6	38840894	38992112	3.93E-09	LCORL
ENSBTAG00000045804^a^	6	38541579	38541674	4.71E-09	novel gene
ENSBTAG00000005932^a^	6	38614370	38672306	8.01E-09	FAM184B
ENSBTAG00000011187^a^	6	37355568	37457493	1.29E-08	FAM13A
ENSBTAG00000019441^a^	6	38603034	38608841	1.53E-08	MED28
ENSBTAG00000032819^a^	1	71072051	71085633	8.44E-08	MUC20
ENSBTAG00000005989^a^	6	38574590	38600027	1.07E-07	LAP3
ENSBTAG00000011973^a^	6	38754415	38754897	1.60E-07	DCAF16
ENSBTAG00000047743^a^	6	41709362	43021626	7.47E-07	KCNIP4
ENSBTAG00000018203^b^	3	26319356	26347752	9.65E-08	CD101
ENSBTAG00000033217^b^	3	16376436	16403812	9.78E-08	TPM1
ENSBTAG00000015147^b^	3	18799612	18810545	5.19E-07	S100A10

Ensemble ID = gene ID name in Ensemble database.

BTA = Bos Taurus Autosome.

Start(bp) = the gene start position (bp) on Ensemble database.

End(bp) = the gene end position (bp) on Ensemble database.

P value = p-values calculated by Fisher’s combination test.

Gene symbol: the corresponding gene name.

**Table 4:** Selected non-overlapping expressed genes for two traits.

Gene(s) Abbreviation	Comments	References
*SLIT2*^*a*^	A genome-wide significant association with cattle carcass weight, broiler chicken cross has been reported. Cellular Function and Maintenance, Skeletal and Muscular System Development and Function.	46, 47, 48
*PAPPA2*^*a*^	Pregnancy associated plasma protein A2 (PAPP-A2) affects bone size and shape and contributes to natural variation in postnatal growth in mice. Researchers found SNP near this PAPPA2 to be associated with postpartum anestrous interval in Tropical Composite cattle.	49, 50, 51
*FAM13A*^*a*^	FAM13A (family with sequence similarity 13, member A) gene, which has been shown to be associated with mastitis in Jersey cows. FAM13A is associated with skeletal muscle mass. The principal determinants of skeletal muscle mass are the muscle fiber number and muscle fiber size.	52,53
*MED28*^*a*^	MED28 is significant association with bovine carcass weight. It is most highly expressed in the bovine pineal gland, lyphoreticular tissue, uterus, abomasum, and expressed in lower quantities in the liver, reticulum, intestine and kidney	54,55
*MUC20*^*a*^	This gene encodes a member of the mucin protein family. Mucins are high molecular weight glycoproteins secreted by many epithelial tissues to form an insoluble mucous barrier.	56,57
*KCNIP4*^*a*^	The KCNIP4 gene (Kv channel-interacting protein 4) has a role in the calcium ion binding, and in potassium and voltage-gated ion channel activity. It has a significant association with yearling weight Canchim Beef Cattle.	58,59
*DCAF16*^*a*^	DCAF16 takes part in the process of protein ubiquitination and acts as Cul4-RING E3 ubiquitin ligase complex in cellular component. Some researchers indicate a significant difference in the expression of the DCAF16 genes in fetal and adult bovine longissimus muscle.	35,60
*CD101*^*b*^	A genome-wide significant association with cattle Milking Speed, pig feed efficiency has been reported. Plays a role as inhibitor of T-cells proliferation induced by CD3. Inhibits expression of IL2RA on activated T-cells and secretion of IL2. Inhibits tyrosine kinases that are required for IL2 production and cellular proliferation, blocking Ca2 + Flux.	61,62
*TPM1*^*b*^	This gene associated with Muscle and Fat Tissues of Native Korean Cattle. Tropomyosin 1 (Alpha) is a member of the tropomyosin family of highly conserved, widely distributed actin-binding proteins involved in the contractile system of striated and smooth muscles and the cytoskeleton of non-muscle cells.	63,64
